# Implementation of Internet-based preventive interventions for depression and anxiety: role of support? The design of a randomized controlled trial

**DOI:** 10.1186/1745-6215-10-59

**Published:** 2009-07-27

**Authors:** Tara Donker, Annemieke van Straten, Heleen Riper, Isaac Marks, Gerhard Andersson, Pim Cuijpers

**Affiliations:** 1 Department of Clinical Psychology, VU University, van der Boechorstraat 1, 1081 BT Amsterdam, The Netherlands; 2 Netherlands Institute of Mental Health and Addiction, P.O. Box 725, 3500 AS Utrecht, The Netherlands; 3 Institute of Psychiatry, King's College London, De Crespigny Park, SE5 8AF, London, UK; 4 Department of Behavioural Sciences and Learning, Swedish Institute of Disability Research, Linköping University, SE-581 83 Linköping, Sweden; 5 Department of Clinical Neuroscience, Karolinska Institutet, SE-171 77, Stockholm, Sweden

## Abstract

**Background:**

Internet-based self-help is an effective preventive intervention for highly prevalent disorders, such as depression and anxiety. It is not clear, however, whether it is necessary to offer these interventions with professional support or if they work without any guidance. In case support is necessary, it is not clear which level of support is needed. This study examines whether an internet-based self-help intervention with a coach is more effective than the same intervention without a coach in terms of clinical outcomes, drop-out and economic costs. Moreover, we will investigate which level of support by a coach is more effective compared to other levels of support.

**Methods:**

In this randomized controlled trial, a total of 500 subjects (18 year and older) from the general population with mild to moderate depression and/or anxiety will be assigned to one of five conditions: (1) web-based problem solving through the internet (self-examination therapy) without a coach; (2) the same as 1, but with the possibility to ask help from a coach on the initiative of the respondent (on demand, by email); (3) the same as 1, but with weekly scheduled contacts initiated by a coach (once per week, by email); (4) weekly scheduled contacts initiated by a coach, but no web-based intervention; (5) information only (through the internet). The interventions will consist of five weekly lessons. Primary outcome measures are symptoms of depression and anxiety. Secondary outcome measures are drop-out from the intervention, quality of life, and economic costs. Other secondary outcome measures that may predict outcome are also studied, e.g. client satisfaction and problem-solving skills. Measures are taken at baseline (pre-test), directly after the intervention (post-test, five weeks after baseline), 3 months later, and 12 months later. Analysis will be conducted on the intention-to-treat sample.

**Discussion:**

This study aims to provide more insight into the clinical effectiveness, differences in drop-out rate and costs between interventions with and without support, and in particular different levels of support. This is important to know in relation to the dissemination of internet-based self-help interventions.

**Trial Registration:**

Nederlands Trial Register (NTR): TC1355

## Background

Depressive and anxiety disorders are highly prevalent among society [1,2] and responsible for high societal costs [3]. Therefore, effective and inexpensive treatments should be made readily available [4]. Despite its effectiveness [5,6], only a small number of people receive psychological treatment by a properly trained mental health professional. Several reasons can account for this, such as long waiting lists, high costs [7] and limited accessibility [4].

Internet usage increased rapidly among the world population by over 300% [8] over the past eight years, with a population coverage of 48% to 78% in the Western countries. As much as 35% to 80% of users consult the internet for health care purposes [9]. This trend creates new opportunities for health care providers to reach their target population, for example via online mental health treatment.

Internet-based self-help interventions are standardized psychological treatments provided online, in which a patient can help him- or herself, either independently or with the help of a (professional) therapist. The support given by a therapist or coach can vary from more personalized extensive written input [10] to minimal contact [11]. Several well-designed randomized controlled trials report that internet-based self-help interventions for mild depression and anxiety are effective in reducing these symptoms [12,13]. It can be expected, therefore, that the use of (guided) self-help through the internet will be increasingly used in the prevention of common mental disorders. Advantages of these kinds of treatments are, for example, the reduced therapist time [14], reduced costs [7] and the ability to reach populations with mood and anxiety disorders who are not reached with more traditional forms of treatment [15].

Although the efficacy of internet-based self-help has been demonstrated sufficiently, it is not clear how these interventions should be offered to the population who can benefit from it. A major issue, for example, is whether it is better to offer the intervention with the support of a coach or if automated programs could work. In case support is necessary, it is not clear which level of support is needed. One of the advantages of a system without a coach is the ease and low costs of implementation, as it does not require a complex and costly structure of professionals. Furthermore, there is virtually no limit as to how many clients can enter the program, since additional clients will not imply additional therapist time [4]. On the other hand, a system organization, in which participants have to be assigned to coaches, will account for high costs and is more difficult to organize, since the coaches have to be trained, have to have sufficient time and have to be paid. There will also be limits on the amount of participants entering the program. However, legal and ethical issues of unguided programs (e.g., disappointment if the treatment fails, and the question of responsibility), the necessary risks of failing to identify cases for whom pure self-help is not enough, and the inability to monitor patients and adjust their treatment when necessary (e.g., in crisis situations) are arguments against unguided programs.

There is evidence that in interventions without a coach, compared to interventions with a coach, the drop-out rate is considerably higher [12]. Moreover, although interventions without a coach can be effective in reducing depressive and anxiety symptoms, they are associated with much smaller effect sizes than interventions in which the user had regular contact with a coach [12]. How large the differences in effectiveness are between interventions with and those without a coach has not been sufficiently studied. Moreover, it has not been examined whether there are differences in drop-out rate and costs between interventions with and without support, and in particular different levels of support. The aim of this study is to examine whether an internet-based self-help intervention with a coach is more effective than the same intervention without a coach in terms of clinical outcomes, drop-out and economic costs. Furthermore, this study examines which level of support by a coach is more effective compared to other levels of support.

## Methods

### Study design

This study is a randomized controlled trial with three active treatments with different levels of support and two control conditions (general support by coach and information only). The three active treatment and control conditions are:

1. *Web-PS*: Brief internet-based **p**roblem-**s**olving on the internet ('self-examination' treatment, see below) without coaching (but with automated emails at regular times).

2. *Web-PS + c*: The same as in 1, but with the possibility for subjects on their own initiative to email a **c**oach asking for support.

3. *Web-PS + C *The same as in 1, but with a coach who will actively approach the subject at regular pre-agreed times (once per week, by email).

4. *C*: No internet intervention, but a **c**oach will approach the subject at regular pre-agreed times (once per week, by email or chat) to discuss problems and give general support (non-specific intervention).

5. *Web-info*: Only information on depression and anxiety (on the internet).

The non-specific intervention condition (condition 4) is added to control for the non-specific effects of coaching. The study protocol, information brochure and informed consent were approved by the Medical Ethics Committee of the VU University Medical Center (registration number 2008-011).

### Inclusion and exclusion criteria

Participants who return the informed consent will be included in the study if they: 1) are 18 years or older; 2) have symptoms of depression and/or anxiety (as defined by scoring above the cut-off of 16 on the Center for Epidemiological Studies Depression scale (CES-D) and eight on the Hospital Anxiety and Depression Scale (HADS); 3) which are not too severe (1 standard deviation above the population mean on the CES-D, cut-off < 39 and/or HADS cut-off <15 [16,17]), 4) have access to a computer with a fast internet connection and 5) have sufficient knowledge of the Dutch language. Excluded are subjects with severe depressive or anxiety disorders (score ≥ 39 on the CES-D; ≥ 15 on HADS), active suicidal plans (≥ 3 SQ suicide) and/or currently under treatment by a mental health specialist. Participants will not be excluded if they are taking prescribed medicine for anxiety or depressive disorders over one month with stable dosage.

### Recruitment

A total of 500 participants with mild to moderate depression and/or anxiety will be recruited from the general population through banners on internet websites (Google, Dutch internet-sites on mental health issues), as well as local newspapers. All advertisements are in Dutch. In choosing the websites specific attention will be paid to include patients with different ethnic backgrounds and socioeconomic groups. In these banners, a link to a website is given which contains information about the study. Patients can leave their names and email address when interested in the study. More extensive information will then be sent by post, together with an informed consent form and a link by e-mail to the online baseline-questionnaire. Subjects who meet the inclusion criteria (as described above) will be randomized to the different treatment arms and will be notified by email. Subjects who are excluded will be notified by email and advised to contact their GP when their symptoms of depression or anxiety are severe (as determined by the CES-D and HADS). For practical reasons, recruitment will take place in batches of 100 participants.

### Randomization

Based on three factors (depression and anxiety scores, prescribed medication for anxiety or depression), participants will be randomized after the baseline measurement. Subjects will be randomized into five groups (the three active treatment conditions and two control conditions) using block randomization with variable sizes. The allocation schedule will be made with a computerized random number generator by an independent researcher and will be unknown to the investigators.

### Interventions

#### Problem-Solving Treatment

The intervention used in this study for conditions 1–3 is a brief, web-based intervention for problem-solving, called "allesondercontrole", based on self-examination therapy (SET [18]). In problem solving therapy (PST), it is assumed that depressive and anxiety symptoms can be caused by practical problems people face in their daily lives. When people can resolve their problems, their symptoms of depression and anxiety will decrease. During PST an individual learns a specific problem-solving procedure in an attempt to resolve their problems' in a structured way [19]. "Allesondercontrole" is expanded with more information, examples, exercise and forms and consists of five weekly lessons. Participants are allowed to use the program 24/7 as long and often as they want during the trial period. See Warmerdam et al. [19] for a more elaborate description of "allesondercontrole". This website is already available (currently only available for research purposes) and proved to be effective in both international and national research for depression and anxiety [13,18-21].

#### Control groups

The participants in control condition four (only coaching, no internet intervention) receive non-directive support by their coach for a maximum of 15 minutes per week. The support consists of non-directive conversation skill techniques based on Client-centered Therapy [22], communication skills [23] and clinical management used in the National Institute of Mental Health (NIMH) treatment for adolescents with depression study (TADS) [24]. The coach is allowed to give general support only, while at the same time avoiding specific techniques from other formal psychotherapeutic interventions. Participants can choose between emailing or chatting with their coach. Participants will receive the "allesondercontrole" intervention in a self-help book-form [25] six weeks after entering the study. The participants in the control condition five only get 24/7 access to a website with general information about depression and anxiety. The participants in this condition are offered the intervention 6 weeks after entering the study.

#### Support

In condition one, automated standardized emails are sent to subjects with additional information regarding how to make the exercises. There will be no support from a coach.

In condition two, the respondent has the option to contact the coach by email for additional support and in condition three, the coach will actively approach the respondents per email, once per week. This will take 10 to 15 minutes per week; total coaching time is estimated at one to 1.5 hours per respondent [13]. Coaching is only meant to give support in working through the self-help method and not aimed at developing a patient-therapist relation. The participants in condition four receive non-directive support by their coach via email or chat. Coaching will be given by Master level students in clinical psychology. Coaches for condition 2 and 3 will receive a training of approximately six hours given by the first and second author of this article. The training will consist of reading the interventions materials themselves, carrying out assignments, and practicing feedback to each other by e-mail. During the training they will also practice with case material and their feedback will be discussed. The training for condition four, non-directive support, will be given by the first author of this article and consists of carrying out assignments and practicing non-directive conversation skills for approximately four hours.

In all five conditions two automated emails will be send to participants reminding them about when they can fill in the post-test measurement. Based on a recent study of Nordin et al. [Nordin S, Carlbring P, Cuijpers P, & Andersson, G: Expanding the limits of bibliotherapy for panic disorder. Randomized trial of self-help without support but with a clear deadline, submitted], we expect participants to adhere better to the program when they are aware of a deadline, in which their symptoms are surveyed.

### Assessments

Primary outcome measures are symptoms of depression and anxiety. Secondary outcome measures are drop-out from the intervention, quality of life, and economic costs. Other secondary outcome measures that may predict outcome are also studied, e.g. client satisfaction and problem-solving skills. Measures are taken at baseline (pre-test), directly after the intervention (post-test, five weeks after baseline), three months later, and 12 months later. All measurements are self report measures and will be administered through the internet.

### Instruments

#### Primary outcomes

##### Depressive symptoms

###### CES-D

The Dutch version of the Center for Epidemiological Studies Depression scale (CES-D [26] has 20 self-rated items; each scored 0–3; total score range is 0–60. Among different populations, the paper-pencil CES-D has acceptable psychometric properties with a cut-off score of 16 (sensitivity: 0.84–1.00, specificity: 0.69–0.90 [27-29]). The Internet CES-D is also reliable and valid with a cut-off score of 22 (Cronbach's alpha: 0.90; AUC: 0.84; sensitivity: 0.94; specificity: 0.62) in an adult population [30].

###### PHQ-9

The nine-item mood module of the Patient Health Questionnaire (PHQ-9 [31]) is used to screen and to diagnose patients with depressive disorders. The 9 items are each scored 0–3, total score range is 0–27. In a review of Wittkampf et al. [32], a sensitivity of 0.77 (0.71–0.84) and a specificity of 0.94 (0.90–0.97) was found for the PHQ-9.

### Anxiety symptoms

#### HADS

The 7-item anxiety subscale (Dutch version) of the Hospital Anxiety and Depression Scale (HADS [33] is used for identifying anxiety symptoms. Cronbach's alpha ranged between 0.81 to 0.84, in different normal and clinical Dutch samples [34]. Item-responses are on a 0 to 3 scale, total score range is 0–21 with higher scores indicating more anxiety.

#### BAI

The 21-item Beck Anxiety Inventory (BAI [35]) assesses anxiety, with a focus on somatic symptoms. Item responses are on a 0 to 3 scale, total score ranges from 0 to 63, with higher scores indicating more anxiety. The BAI has high internal consistency and demonstrated good convergent and divergent validity [36,37].

### Secondary outcomes

#### Quality of life

The 5-item self-report Euroqol [EQ; 38] measures health-related quality of life and consists of five dimensions (mobility, self-care, main activity, pain and mood), each of which is rated as causing 'no problems', 'some problems', or 'extreme problems'. The EuroQol valuations appear to have good test-retest reliability [39]. Brazier et al. [40] found considerable evidence for the construct validity of the EQ. There was substantial evidence of EQ being less sensitive at the ceiling (i.e. low levels of perceived ill-health) and throughout the range of health states.

#### Health care utilization

A health service uptake and production loss due to illness, which produces economic costs, is measured with the iMTA Questionnaires on Costs Associated with Psychiatric Illness (TiC-P [41]). This structured interview gives an indication of direct and indirect costs of the intervention and consists of two parts: (i) the amount of medical care received by the participants and (ii) work productivity.

#### Working alliance inventory (WAI)

The Working alliance inventory (WAI [42]), measuring work alliance, is a 36-item self-report questionnaire and makes use of Likert response scale from never to always. Good construct validity and good to high internal consistency was found in a study of Horvath and Greenberg [42], its construct validity is supported in other studies in which the WAI was compared to other working alliance instruments [43].

Only the 21 questions which were relevant to our type of study (role of support) will be used, which are: questions 1–3, 5, 6, 8–10, 12, 17–21, 24, 25, 27–29, 32–33. All words regarding "nurse" or "nurse-treatment protocol" will be changed to "coach" and "treatment protocol". This questionnaire will only be administered to those in condition 2, 3 and 4 (with a coach).

#### Client satisfaction

The Dutch version of the Client Satisfaction Questionnaire (CSQ-8 [44,45]) is used to assess global patient satisfaction. De Brey [45] found a high internal consistency (Cronbach's α = 0.91), similar to the original English version (Cronbach's α = 0.93). The 8-item self report questionnaire scale response options are from 1 to 4, total score ranges from 8 to 32.

#### Mastery

The 7-item Mastery Scale [46] ranges from 1 to 5, total scale score 7–35. A high score (internal mastery) indicates that someone has the feeling to be in control of situations. A low score (external mastery) indicates that someone has the feeling that things are out of their control. The questionnaire has good psychometric properties [46].

#### Expectancy

The Credibility/Expectancy Questionnaire of Devilly and Borkovec (CEQ [47] is used to measure expectancy for change and treatment credibility. These two factors have been found to be stable across different populations, with high internal consistency within each factor and very high test-retest reliability [48]. It comprises six questions, four on "thinking" and two on "feeling." On the "thinking" questions, three are rated on a Likert-type scale from 1 to 9 and the fourth is rated from 0 to 100%.

#### Web Screening Questionnaire for common mental disorders (WSQ)

To quickly detect if participants have symptoms of depression or anxiety, we administer the WSQ simple version. Sensitivity of the WSQ ranged from 0.60 to 1.00 while specificity varied between 0.44 and 0.77 [49].

#### Personality disorder

The 8-item Standardized Assessment of Personality Abbreviated Scale (SAPAS [50] Moran et al., 2007) is used to screen for personality disorder. Each item is dichotomously scored (0–1), total score ranges from 0 to 8. A score of 3 on the screening interview correctly identified the presence of DSM-IV personality disorder in 90% of participants. The sensitivity and specificity were 0.94 and 0.85 respectively [50].

#### Motivation

To measure the motivation of participants for self-help therapy, we use three questions of the screening questionnaire (SQ), a quick self-report developed by Marks et al. [51]. Likert-scale response options are used, total score ranges from 0 to 8.

#### Suicidal intentions

To measure suicidal intentions of participants we use one question of the screening questionnaire (SQ), a quick self-report developed by Marks et al. [51]. Likert-scale response options are used, total score ranges from 0 to 3, cut off score is above 3.

#### Problem solving skills

The 52-item Social Problem-Solving Inventory-Revised (SPSI-R; [52]) is used for measuring problem solving skills. Three of the five scales ((Positive problem Orientation (PPO; five items), Negative Problem Orientation (NPO; 10 items), and Avoidance Style (AS; seven items) showed to be sensitive for change [Warmerdam EH, van Straten A., Jongsma J, Twisk J, Cuijpers P: Online cognitive behavioral therapy and problem-solving therapy for depressive symptoms: Exploring mechanisms of change, submitted] and were therefore included in the study. Good internal consistency and test retest reliability are reported for all SPSI-R subscales within the manual [53].

#### Non-response

The Internet Intervention Adherence Measure [54] will be used to identify obstacles that interfered with the patient completing the program. Obstacles are categorized as Internet/computer/technical issues, personal/family issues, intervention-general issues, and intervention-specific issues. Patients are asked to respond to the 35 items on a 3-point scale from 1 to 3, indicating whether that obstacle had "no part," "a little part," or "a major part" in why they stopped using the program.

#### Other questions: demographic variables, time, currently under treatment

We will add demographic variables, location were participants rated the questionnaires, medication use and whether participants receive treatment by a mental health specialist.

For an overview of the instruments see Table 1.

**Table 1:** Overview of questionnaires

		**Time of measurement**			
**Self rated questionnaires**	**Aim**	**T0** **Baseline (pre-test)**	**T1** **Post-test**	**T2** **Follow-up** **(3 months)**	**T3** **Follow-up (12 months)**
CES-D	Symptoms of depression	X	X	X	X
PHQ-9	Symptoms of depression	X	X	X	X
HADS	Symptoms of anxiety	X	X	X	X
BAI	Symptoms of anxiety	X	X	X	X
EuroQol	Quality of life	X	X	X	X
TIC-P	Health service uptake and production losses	X	-	X	X
Mastery scale	Mastery	X	X	X	X
CSQ-8 (condition 1–4)	Client satisfaction with treatment	-	X	-	-
WAI (condition 2–4)	Client satisfaction with coach	-	X	-	-
CEQ	Expectancy and treatment credibility	X	-	-	-
SQ	Motivation	X			
SAPAS	Personality disorder	X	-	-	-
WSQ	Common mental disorders	X	-	-	-
SPSI-R	Problem solving skills	X	X	X	X
Internet-Intervention Adherence Survey	Non-response, attrition	-	i.a.	i.a.	i.a.
Various	User characteristics	X	-	-	-

*i.a.: if applicable

### Sample size

The effects of the intervention on symptoms of depression and anxiety are the primary outcome measures and these are used as starting point for the power calculations. We assume that we want to be able to show differences between interventions and the control conditions with a standardized effect size (Cohen's *d*) of 0.35 [13]. Smaller effect sizes are considered to be not relevant from a clinical point of view. Assuming an alpha of 0.05 and a statistical power (1-Beta) of 0.80 in a one-tailed test, we need 100 respondents in each of the conditions. Overall, we need to recruit 500 respondents in total.

### Statistical analysis

All analyses will be conducted according to the intention-to-treat principle. All participants who have been randomized will be included in the analyses examining the differential effects of the interventions. Missing data will be imputed using regression imputation. To examine differences between the conditions, we will use multiple regression analyses with symptoms of depression and anxiety as dependent variables and d the treatment dummy as predictor. By using multiple regression analyses, we can correct for possible confounders. All analyses will be conducted using SPSS for Windows, version 15.

#### Economic analyses

Economic costs of the intervention (internet application, costs of the coaches) will be calculated per participant, and both in money and hours working time. We will also conduct a true economic evaluation. The pertinent cost-data will be collected in the experimental and control conditions at baseline and at follow-up with help of the TIC-P [41]. The incremental cost-effectiveness ratio (ICER) will be calculated. Its uncertainty will be graphically represented in the ICER planed with help of the bootstrap method (with 2,500 bootstrap replications). Finally, the ICER acceptability curve will be plotted for several ceilings in the willingness to pay for a Quality-Adjusted Life Year (QALY) gained. Sensitivity analyses will be carried out to ascertain the robustness of the findings under different scenarios, e.g. under varying values of key-variables.

## Discussion

This study examines whether an internet-based self-help intervention for depressive and anxiety symptoms with a coach is more effective than the same intervention without a coach in terms of clinical outcomes, drop-out and economic costs. A further objective is to examine which level of support by a coach is more effective compared to other levels of support. It is important to know whether guided (and which level of guidance) or unguided self-help results in the optimal trade-off between reduction of clinical symptoms, drop-out and economical costs, as this will have a huge impact in the dissemination of internet-based self-help interventions. A system without a coach is easy and cheap to implement, does not require a complex and costly structure of professionals and there are no limits to how many clients can enter the program. On the other hand it can be much less effective and doubtful from a practical and ethical point of view. This study aims to provide more insight into the clinical effectiveness, differences in drop-out rate and costs between interventions with and without support, and in particular different levels of support.

This study has several limitations. First, there could be selective drop-out in the non-internet-intervention groups (control condition 4: support only; control condition 5: only information). In the analysis, we will examine whether this happened. Furthermore, we will make use of intention-to-treat analysis, in which missing values are estimated with different techniques (e.g., multiple regression, multiple imputation). Second, the control group receives information only, which can be effective in reducing symptoms of depression and anxiety, although effect sizes are small [11]. Third, psychometric properties of some of the questionnaires used in this study are not yet tested for online use, although this can be different from its paper-pencil versions [55]. One of the reasons is that people sometimes disclose more sensitive information in computer-based compared to face-to-face interviews [56]. A final limitation of this study concerns the sole use of self-rated instruments as outcome measures and the lack of a diagnostic interview; hence we have no knowledge whether subjects meet the criteria for a DSM-IV diagnosis of depression or anxiety. The lack of the gold standard of diagnosing makes it difficult to compare with other studies. However, since this preventive intervention is aimed to be applicable and accessible for a broad population of people from the community with clinically relevant symptoms of depression and anxiety [19], a formal diagnosis will not be necessary for participants to enter the program.

One of the strengths of this study concerns the formal evaluation of the cost effectiveness for the different levels of support given in the intervention, which has not been studied sufficiently previously [4]. Furthermore, by including the condition in which no intervention is given but only support of a coach, we will be able to examine the non-specific effects of coaching. Finally, external validity is a strong aspect of this study, since specific attention is given in the recruitment process to include patients with different ethnic backgrounds and socioeconomic groups.

## Abbreviations

BAI: Beck Anxiety Inventory; C: Coach; CES-D: Center for Epidemiological Studies Depression scale; CSQ: Client Satisfaction Questionnaire; CEQ: Credibility/Expectancy Questionnaire; DSM-IV: Diagnostic Statistical Manual, 4^th ^edition; Euroqol [EQ HADS: Hospital Anxiety and Depression Scale; GP: General Practitioner; ICER: incremental cost-effectiveness ratio; *i*MTA: Institute for Medical Technology Assessment; NIMH: National Institute of Mental Health; PHQ-9: Patient Health Questionnaire; PST: problem solving therapy; QALY: Quality-Adjusted Life Year; SAPAS: Standardized Assessment of Personality Abbreviated Scale; SET: self-examination therapy; SPSS: Statistical Package for the Social Sciences; SQ: Screening Questionnaire; TADS: treatment for adolescents with depression study; TiC-P: Trimbos/iMTA questionnaire for Costs associated with Psychiatric Illness; WAI: Working alliance inventory; Web-PS: internet-based **p**roblem-**s**olving; WSQ: Web Screening Questionnaire for common mental disorders

## Competing interests

The authors declare that they have no competing interests.

## Authors' contributions

PC and HR obtained funding for the study. All authors contributed to the design of this study. PC and AvS made the Internet-based PST-intervention "Allesondercontrole". TD drafted the manuscript. All authors contributed to the further writing of the manuscript. All authors read and approved the final manuscript.
